# Beyond Pristine Metal–Organic Frameworks: Preparation of Hollow MOFs and Their Composites for Catalysis, Sensing, and Adsorption Removal Applications

**DOI:** 10.3390/molecules28010144

**Published:** 2022-12-24

**Authors:** Xiaoqian Zha, Xianhui Zhao, Erin Webb, Shifa Ullah Khan, Yang Wang

**Affiliations:** 1 School of Chemistry and Chemical Engineering, Yangzhou University, Yangzhou 225002, China; 2 Environmental Sciences Division, Oak Ridge National Laboratory, 1 Bethel Valley Road, Oak Ridge, TN 37830, USA; 3 The Institute of Chemistry, Faculty of Science, University of Okara, Renala Campus, Punjab 56300, Pakistan

**Keywords:** hollow metal–organic framework, catalysis, sensing, adsorption

## Abstract

Metal–organic frameworks (MOFs) have been broadly applied to numerous domains with a substantial surface area, tunable pore size, and multiple unsaturated metal sites. Recently, hollow MOFs have greatly attracted the scientific community due to their internal cavities and gradient pore structures. Hollow MOFs have a higher tunability, faster mass-transfer rates, and more accessible active sites when compared to traditional, solid MOFs. Hollow MOFs are also considered to be candidates for some functional material carriers. For example, composite materials such as hollow MOFs and metal nanoparticles, metal oxides, and enzymes have been prepared. These composite materials integrate the characteristics of hollow MOFs with functional materials and are broadly used in many aspects. This review describes the preparation strategies of hollow MOFs and their composites as well as their applications in organic catalysis, electrochemical sensing, and adsorption separation. Finally, we hope that this review provides meaningful knowledge about hollow-MOF composites and their derivatives and offers many valuable references to develop hollow-MOF-based applied materials.

## 1. Introduction

A novel category of polyporous substances called MOFs are composed of coordinately bound metal ions and organic ligands. They possess unique structural features, a substantial surface area, a tunable pore diameter, unsaturated metal coordination nodes, and abundant organic ligands [1]. Over the past twenty years, MOFs of various MIL [2,3], UIO [4,5], and ZIF series [6,7] have been prepared, and more than 20,000 have been reported so far. MOFs have been found to possess a good amount of redox sites and can be utilized in a variety of domains such as catalysis, electrochemical sensing, adsorption, removal, and drug delivery [8,9,10,11]. The shape of an MOF can be easily modified by changing parameters during synthesis. Therefore, there has been a lot of focus and effort put towards the applications of MOFs and their structural regulation.

The hollow structure is an advanced material with an adjustable inner cavity and a thin shell. It possesses plenty of merits, such as a large, closed chamber, low density, substantial surface area, and a solid framework [12,13]. When compared to solid MOFs, hollow MOFs retain their inner cavity and graded porosity in addition to the inherent advantages of original MOFs. The synergistic relationship between the cavity and the MOF’s skeleton enables a hollow MOF to have a faster mass-transfer rate, lower density, and more abundant active sites. Regarding catalysis, the mass-transfer resistance of a guest molecule in an MOF is greatly reduced by the cavity inside the hollow MOF, thereby enhancing the catalytic performance. In addition, the morphology of hollow MOFs result in greater tunability, such as the double–shell, triple–shell, and yolk–shell structures of hollow MOFs [14,15,16]. Abbreviations used throughout this review article are in Table 1.

Much has been done to explore hollow MOFs with more complex architectures. For example, the hollow, double-shell structure Fe_3_O_4_@Pd/ZIF–8@ZIF–8 was prepared by Zhong and his group for a highly efficient catalytic hydrogenation reaction with excellent repeatability and selectivity [17]. However, the application of hollow MOFs still presents great challenges. MOFs’ inherent weaknesses, such as their insufficient stability and weak electrical conductivity, severely constrain their applications. The stability of an MOF mainly depends on the interaction between the metal source and the organic ligand. If MOFs are exposed to a moist environment, the interaction will be destroyed due to the substitution of metal–containing clusters by water or other nucleophiles, resulting in the collapse of the skeleton. In addition, poor conductivity is one of the important aspects that limit MOF application in the field of electrochemical sensing. Although solid MOFs have a porous structure, they still prevent the long–distance charge transport of spatially separated redox components in the material to a large extent, resulting in the output of detection signals. Therefore, efforts have been made to overcome these problems by compounding various materials with hollow MOFs. For instance, many conductive materials such as metal nanoparticles [18,19], polymers [20], enzymes [21,22], reduced graphene oxide [23], and carbon nanotubes [24], etc., have been combined with hollow MOFs to enhance their mechanical performance, electrical conductivity, and catalytic activity.

In recent years, the synthesis and application of hollow MOFs have generated great interest and numerous breakthroughs have been achieved. This article summarizes the fabrication strategies of hollow MOFs and introduces some of the latest research progress. We mainly focus on the research frontiers of hollow MOFs in sensing, chemical catalysis, and adsorption removal.

## 2. Synthetic Strategies of Hollow MOFs and Their Composites

**Scheme 1:**
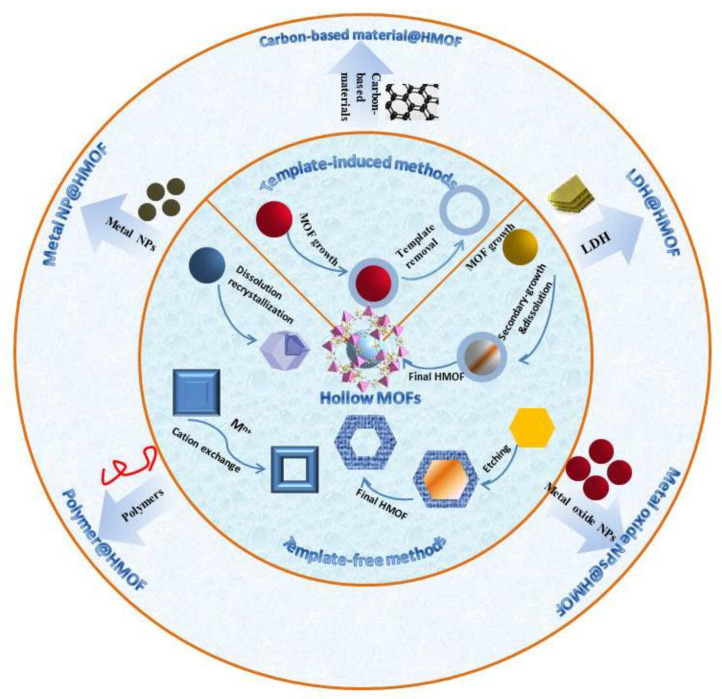
The synthetic strategies of hollow MOFs and their composites.

When compared to traditional, solid MOF, a hollow MOF has a unique cavity structure. Therefore, the preparation of a hollow MOF requires different methods and techniques. So far, there are many methods available for creating hollow MOFs. There are two types that can be distinguished based on the presence of templates: template-induced and template-free methods (Scheme 1).

### 2.1. Template-Induced Methods

A large number of templates are available for template-induced methods, such as SiO_2_, polymers, surfactant, and metal oxides. The main process for preparing hollow MOFs consists of three parts: (1) establishing templates, (2) growing MOFs on templates, and (3) removing templates. The formation and removal of templates have a significant impact on the hollow structure.

Due to the stringent requirements for creating hollow MOFs, the selected templates should meet the conditions of easy preparation and gentle removal as much as possible, otherwise the original morphology and hollow structure of the MOFs will be difficult to maintain. In addition, templates are usually pretreated to ensure strong interactions between MOFs and the templates by forming functional groups or changing the surface charge and polarity so that the MOF can bind tightly to the template.

#### 2.1.1. Hard-Template-Induced Methods

**Figure 1:**
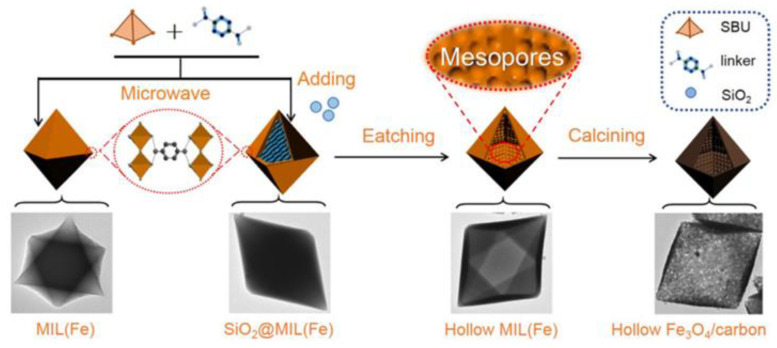
Scheme on the formation of a hollow MIL (Fe) and its derivatives. Reprinted with permission from Ref. [25]. Copyright 2019, Elsevier.

Hard templates, including silicon dioxide [25], polymers [26], and metal oxides [27], etc., usually have well–defined shapes that are easily removed when MOFs are grown on their surfaces, resulting in hollow structures (Figure 1). Meanwhile, the original shape is not damaged. There are three main steps to using hard templates. 

First, a template of fixed shape and size is prepared. Its surface is then modified. By adding a precursor solution, the MOF shells will grow on the exterior of the template and the inner part can be eliminated. Finally, an MOF with a hollow structure is obtained. Among various hard templates, the polymers can be removed under mild conditions, so they are frequently used in the construction of hollow MOFs.

**Figure 2:**
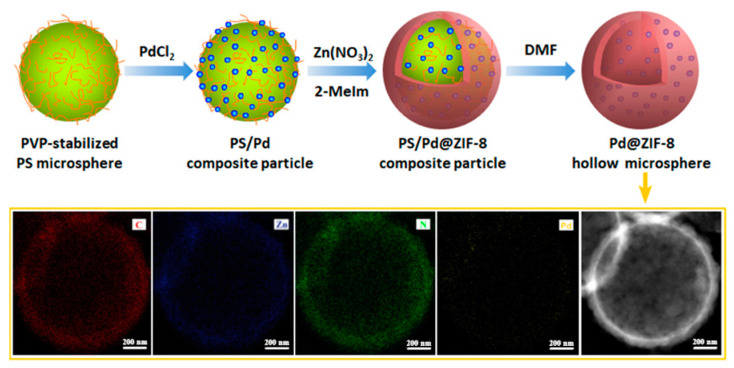
Schematic illustration of the fabrication process of a Pb@ZIF–8 hollow microsphere. Reprinted with permission from Ref. [28]. Copyright 2020, American Chemical Society.

Zhao et al. successfully prepared a hollow ZIF–8 loaded with Pd by using a polystyrene sphere as a template [28]. Due to the reducibility of polyvinyl pyrrolidone and its affinity with Pd, nanoparticles can be immobilized on PS spheres without using external reducing agents. After adding Zn(NO_3_)_2_ and 2–methylimidazole, ZIF–8 developed outside the template due to the coordination connection between the Zn^2+^ and C=O group of the imidazole ring. According to Figure 2, the template was removed by immersing the PS/Pd@ZIF–8 in DMF. The hollow structure was obtained after multiple washings. Additionally, Srinivasapriyan prepared a hollow, sandwich–structure MIL–100(Fe)@Pt@MIL–100(Fe) using a PS sphere as a template [29]. The core–shell structure of PS@MIL–100(Fe) was obtained five growth cycles later. The pre-synthesized Pt nanoparticles were then mixed with PS@MIL–100(Fe) by stirring and adsorbed outside the MOF. The formation route of MIL–100(Fe) was further repeated to obtain a sandwich structure. After soaking in DMF, a hollow sandwich structure of MIL–100(Fe)@Pt@MIL–100(Fe) was ultimately achieved. With the exception of polymers, metal oxides are also frequently devoted to fabricating hollow MOFs. For example, Tsung et al. demonstrated the loading of a layer of Cu_2_O outside Pd as a sacrificial template and then caused the formation of ZIF–8 shells outside the Cu_2_O [30]. Surprisingly, Cu_2_O could be etched by the released protons as the ZIF–8 grew and obtained yolk–shell structured Pd@ZIF–8. A large cavity existed between the Pd nanoparticles and the ZIF–8 shell. In this method, Cu_2_O was used as sacrificial template. On one hand, it could be etched by protons released during the ZIF–8 growth without extra etchant. Moreover, the absence of a capping agent on the Cu_2_O surface gave the ZIF–8 a clean surface to grow on. This method of covering the surface of different metal nanoparticles with Cu_2_O as a sacrificial template has also been extended [31].

The hard-template method can intuitively obtain hollow MOFs, and the morphology and size of a hollow MOF can be effectively regulated by adjusting the template. Nevertheless, the hard–template method also has many disadvantages. The selected template usually needs to be preprocessed before it can be used. Another important issue is that an extra etchant needs to be used to remove the template, and the shape of the hollow MOF is significantly influenced by the etching conditions. Under severe etching conditions, hollow structures may collapse. Therefore, mild etching conditions are critical when choosing a template.

#### 2.1.2. Soft-Template-Induced Methods

**Figure 3:**
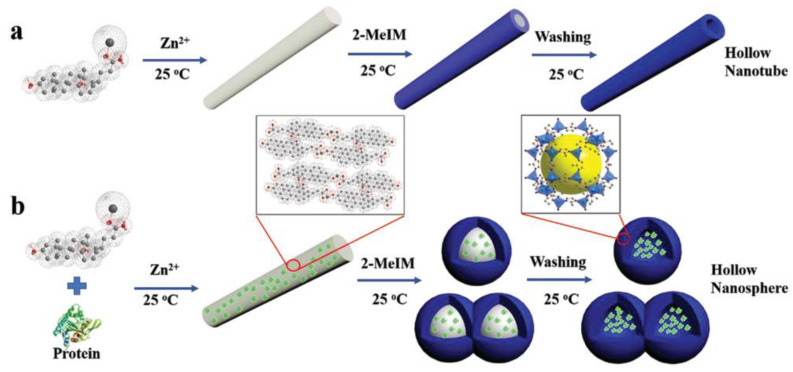
Schematic diagram of the formation process of hollow–MOF tubes and spheres. Reprinted with permission from Ref. [33]. Copyright 2019, WILEY-VCH Verlag GmbH.

Soft templates such as surfactants [32] and hydrogels [33] are widely used in the construction of hollow MOFs (Figure 3). When compared to hard templates, the removal requirements of soft templates are more moderate and can better ensure the integrity of the hollow structure. Surfactants such as alkyltrimethylammonium bromide (C_n_TAB) and sodium dodecyl sulfate (SDS) are often chosen as soft templates due to their unique amphiphilic properties. Zeng et al. synthesized hollow ZIF–67 by using C_n_TAB (n = 12, 14, 16) as soft templates [34]. Alkyltrimethylammonium bromide, a typical class of amphiphilic anionic surfactants, can form [Co(HmIM)(MIM)(L_2_)]^-^ with cobalt ions in an aqueous solution. Due to the thermally adverse effects of exposing the lengthy hydrocarbon chains of C_14_TA^+^ to water, vesicles with a bilayer structure of stacked hydrophobic chains may form. To equalize the overall charge, [Co–complex] and various anions were used around the C_n_TA^+^ ammonia head group. At nucleation sites, [Co–complex] was concentrated on the exterior of the vesicles to induce the development of ZIF–67 shells. The resulting material was treated with DMF and methanol and yielded ZIF–67 hollow spheres. By taking advantage of this method, the author extended this strategy to another hollow–MOF synthesis and successfully prepared HKUST–1 hollow spheres. Lei et al. prepared hollow ZIF–8 using SDS as a template and encapsulated the anticancer drug 10–Hydroxy Camptothecin in the internal cavity of ZIF–8 to realize drug delivery [35]. During preparation, when the concentration of SDS exceeded the critical micelle concentration (CMC), micelles (which were externally hydrophilic and internally hydrophobic) were formed. Zinc nitrate was then added, and a p-p interaction between Zn^2+^ and O atoms resulted in the attachment of zinc ion to the hydrophilic groups. After the addition of 2–methylimidazole, organic ligands and zinc ions rapidly formed ZIF–8 crystals outside the micelles, forming a cavity.

**Figure 4:**
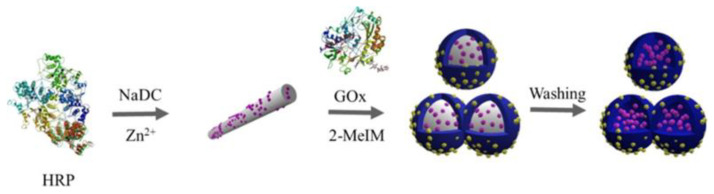
Fabrication of HRP@H–ZIF–8–GOx. Reprinted with permission from Ref. [36]. Copyright 2019, American Chemical Society.

In addition, biological reagents are also utilized as soft templates. For instance, Jiang et al. successfully obtained hollow ZIF–8 using a soft template made of a metal–sodium deoxycholate (NaDC) hydrogel and immobilized glucose oxidase and horseradish peroxidase in its shell and cavity, respectively [36]. As can be seen in Figure 4, NaDC interacted with Zn^2+^ to form hydrogel fibers in the aqueous solution, which served as a soft template for fabricating hollow ZIF–8. After 2-methylimidazole was added, it combined with zinc ions to generate ZIF–8. The template was gradually dissolved, and the hollow-structure ZIF–8 was obtained by washing. Although hollow MOFs can be obtained using the soft-template method, this method cannot always strictly control the size and shape of the products because most soft templates are aggregates formed by amphiphilic molecules.

### 2.2. Template-Free Methods

**Figure 5:**
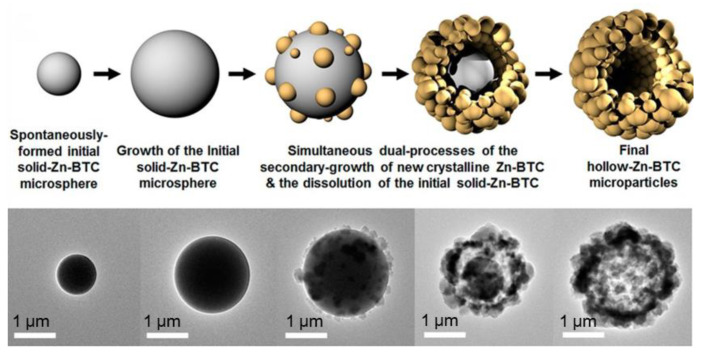
The formation process of hollow–Zn–BTC. Reprinted with permission from Ref. [37]. Copyright 2015, American Chemical Society.

The template-free method can self-assemble into hollow structures by the Ostwald ripening principle, the Kirkendall effect, the surface-energy-driven principle, and the partial-etching method without adding templates (Figure 5) [37,38,39].

#### 2.2.1. Ostwald Ripening Principle

Ostwald ripening is a simple method for preparing hollow materials in which the energy of smaller crystal particles is higher than that of larger particles. As the thermodynamic system continuously releases energy, the smaller particles gradually dissolve and diffuse into the larger particles, forming a hollow structure. Wang et al. successfully synthesized hollow microspheres (Fe–FC–HCPS) [40]. The inner crystallites of the solid aggregates are small and not dense, and these inner crystallites diffuse into the larger outer crystallites in large numbers, thus forming a hollow structure without any additional reagents or templates. Under solvothermal conditions, through the coordination reaction between FeCl_3_ and H_2_FcDC, a large number of small, coordinated, solid-polymer microcrystals were formed in the solution and rapidly aggregated into larger, solid, spherical particles, reducing surface energy.

#### 2.2.2. The Kirkendall Effect

The Kirkendall effect refers to the defect generated during the diffusion of two metal ions at different rates and is also a preparation method for hollow nanoparticles. For the first time, a new method was proposed by Jiang et al. to prepare hollow Au/Zn–MOFs under mild conditions based on the ion–exchange reaction and the Kirkendall effect [41]. After adding Au^3+^, core–shell structure nanoparticles with ZIF–8 as a core and a Au/Zn–MOF as a shell were formed. Due to the imbalance of ion–exchange diffusion between Au^3+^ and Zn^2+^ and the diffusion rate of Zn^2+^, the diffusion out through the shell was higher than the inward diffusion rate of Au^3+^, leading to the creation of Kirkendall holes and hollow structures. Similarly, Ge et al. successfully prepared prismatic, hollow ZIF–67 by using the Kirkendall effect [38]. Firstly, Co–urea NPs were synthesized as a solid precursor and, after 2–methylimidazole was added, Co^2+^ diffused outward from the solid precursor and coordinated with 2-methylimidazole to form the ZIF–67 shell. A hollow structure was ultimately formed due to of the varying diffusion rates of anions and cations.

#### 2.2.3. Surface-Energy-Driven Mechanism

**Figure 6:**
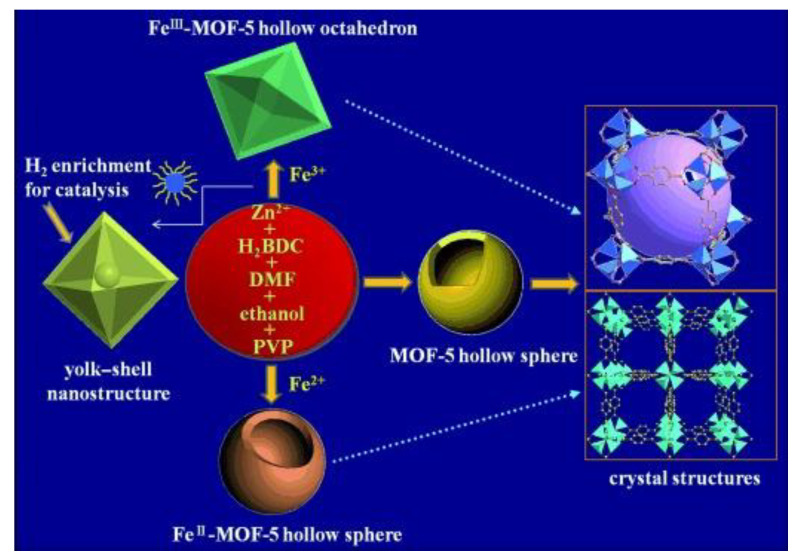
Proposed formation process of different MOF hollow nanocages. Reprinted with permission from Ref. [43]. Copyright 2014, Wiley-VCH Verlag GmbH.

The surface-energy-driven mechanism has been utilized in the preparation of many hollow MOFs. For example, Wang et al. reported that hollow nanocubes made of hierarchic Zn/Ni–MOF–2 nanosheets could be synthesized by employing 1,4–benzenedicarboxylic acid, nickel, and zinc ions and a mixture of N,N–dimethylacetamide (DMAC) and ethanol [42]. The formation of a hollow structure includes three stages; namely, the initial in situ formation of the Zn/Ni–MOF–5, the mixed phase of the Zn/Ni–MOF–2 and Zn/Ni–MOF–5, and the final formation of a hollow Zn/Ni–MOF–2. Similar to the way that inorganic nanocrystals dissolve and then reform, coordination bonds between carboxylic acids and Zn^2+^/Ni^2+^ are broken and then reform during the process of structural transformation. By varying the amount of metal ions added, they found that a Zn–MOF–5 was formed without the presence of a Ni precursor. However, in the absence of a Zn precursor, there were almost no solids because the coordination between the carboxyl group and the zinc ion is better than that of the nickel ion. In addition, a significant part in the creation of hollow MOFs was also performed by the DMAC/ethanol mixed solvent. DMAC can dissolve H_2_BDC and facilitate its deprotonation. Similarly, the same group fabricated a series of FeIII–ICP, FeIII–MOF–5, FeII–MOF–5, and MOF–5 hollow structures using the surface–energy–driven mechanism (Figure 6) [43]. Zn and Fe ions were chosen as the source of metal, PVP was added as a stabilizer, and DMAC (or DMF)/ethanol was used as a mixed solvent. Due to the high concentration of precursors, concave octahedrons were formed during the initial stage of particle formation, which rapidly nucleated and aggregated in the solution, reducing the surface energy. In this reaction, the concentrations of Zn^2+^/Fe^2+^ and organic ligands decreased, and the concave octahedron finally developed into a hollow octahedron with less surface energy through a mass-transfer process from the inside out.

#### 2.2.4. Partial-Etching Method

**Figure 7:**
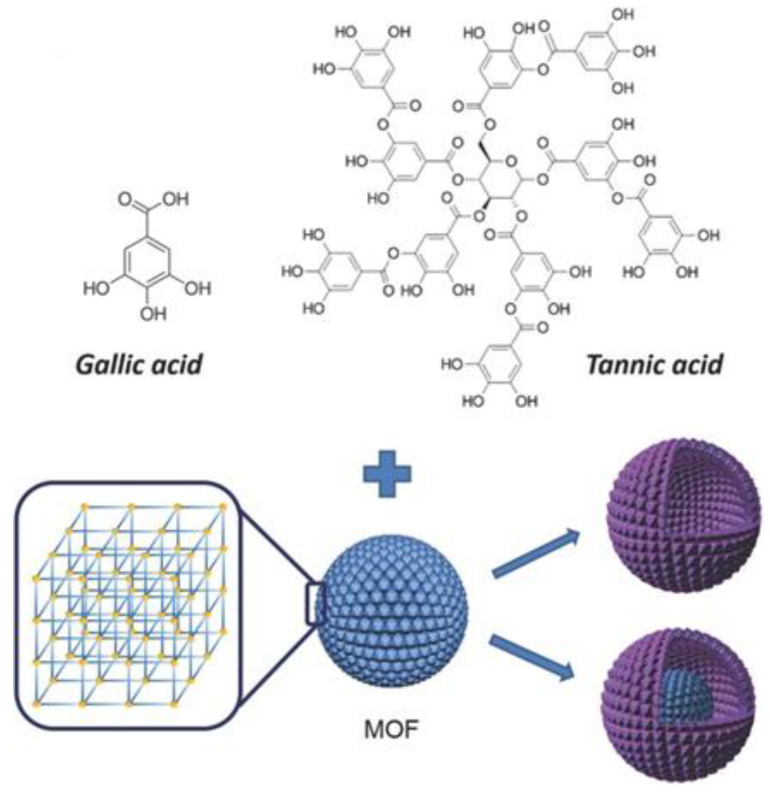
Schematic diagram of the synthesis of the structural formula of gallic acid and tannic acid, and the statement of the eroded process to construct the cavity. Reprinted with permission from Ref. [45]. Copyright 2016, WILEY-VCH Verlag GmbH.

In the partial-etching method, hollow MOFs can be obtained by post–processing the pre-synthesized MOFs. Based on the various etching mechanisms, they can be separated into selective etching and competitive–coordination etching. Selective etching is achieved based on the difference in internal and external structural stability of pre–synthesized materials. A hollow structure is formed because the internal structure is more unstable and can be easily etched by reagents such as acid or alkali (whereas the outer surface is more stable). Liu et al. successfully prepared a bimetallic MIL–101 in an aqueous solution by using two metal salts as metal sources and terephthalic acid as the organic ligand. The MOF was then etched with glacial acetic acid under heating to obtain a hollow MOF with a mesoporous structure [44]. By adjusting the eroded parameter, hollow mesoporous structures possessing various cavity dimensions and shell thicknesses were synthesized. In some MOFs, the stability of internal and external structures is relatively uniform, and the selective etching cannot achieve a precise etching of the interior, leading to the overall structure’s collapse. To address this issue, a surface protectant is used to keep the exterior stable, to stabilize the unevenness of the inner part and the surface, and to achieve precise interior etching. For instance, Hu and his partners synthesized a hollow ZIF–8 using tannic acid (TA) as protectant and etching agent (Figure 7) [45]. The large size of the tannic acid molecule, which is coated on the exterior of the ZIF–8, caused the ZIF–8 to transform from a hydrophobic to hydrophilic structure, promoting H^+^ to diffuse into the interior of the ZIF–8 and achieving the effect of interior etching. Meanwhile, as a protective agent, TA forms a hard shell outside the ZIF–8 to prevent the exterior of the ZIF–8 from being etched. Gallic acid (GA) can also be applied as a protective agent and etching agent, but it differs from TA in that the hollow ZIF–8 shell etched by GA consists of a flake structure, probably because GA has a higher acidity than TA and the structure obtained by GA is not very uniform. Partial etching has better advantages in preparing hollow MOFs. However, due to the relatively weak structural stability of MOFs, the use conditions and acid concentration should be considered in the selection of an etching agent to avoid damaging the original structure of the MOF during the etching process. In the competitive-coordination etching method, due to the weak coordination of precursors, the original coordination of metal ions with organic linkers is often destroyed by external competitors, causing dissolution and recrystallization. Li et al. used 2,5–dihydroxy terephthalic acid (H_4_DOBDC) as a competitive reagent to transform a ZIF–67 into a Co–MOF–74 with a hollow structure using the solvothermal method [46]. First, the pre-synthesized ZIF-67 was reacted with H_4_DOBDC under solvothermal conditions. At the beginning of the reaction process, some areas of the ZIF–67′s surface were eroded by the weakly acidic H_4_DOBDC. Some of the Co^2+^ ions diffused into the solution area near the surface of the ZIF–67, and some Co^2+^ ions were found on the surface. Next, the exposed Co^2+^ ions acted as a nucleation site to react with the DOBDC^4−^, and an MOF–74 began to grow. As the response proceeded, the outer layer of the ZIF–67 was encircled by the MOF–74, forming a MOF–74 shell. Therefore, when the relative content of the H_4_DOBDC decreased, a composite material was created that consisted of a ZIF–67 core and an MOF-74 shell. Along with the increase of the relative content of H_4_DOBDC, a ZIF–67 can be completely transformed into an MOF–74 with a single-shell hollow structure. Through this method, ZIF–67s with different sizes and morphologies can be transformed into Co–MOF–74s with hollow structures and corresponding sizes and morphologies.

In addition to the above methods without templates, the two–phase interface method also does not require a template. In the two–phase interface method, two insoluble phases are used to form the interface, and the precursors of the MOFs are confined within the interface to grow into a thin layer. According to the difference of the two phases, it can be categorized as a gas–liquid, liquid–liquid, or solid–liquid interface. In the gas–liquid interface method, metal ions and ligands are distributed in the liquid phase to provide an interface for the growth of MOFs by dispersing discrete bubbles or droplets. Zhang et al. first proposed a CO_2_–ionic liquid interface method to synthesize hollow Zn–BTC [47].

**Figure 8:**
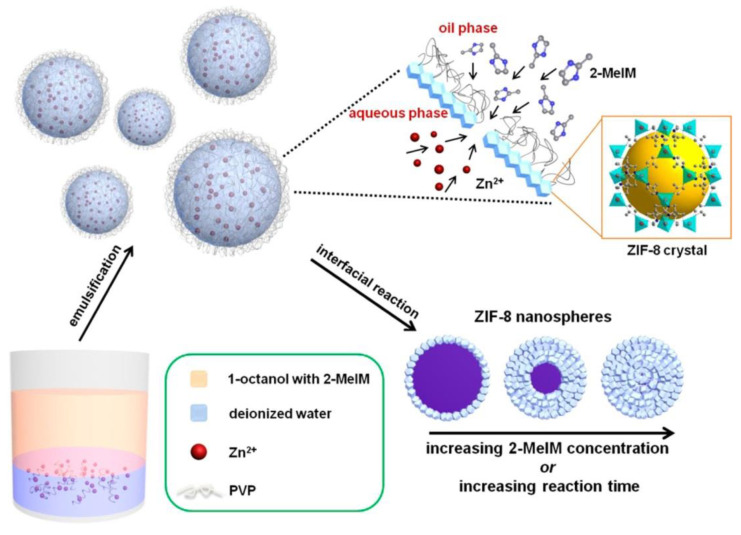
Schematic diagram for the synthesis of a hollow ZIF-8. Reprinted with permission from Ref. [55]. Copyright 2014, American Chemical Society.

By adding CO_2_ and MOF precursors into the ionic liquid phase under stirring, the ionic liquid has a different solubility to both, and bubbles generated when CO_2_ is introduced provide a large number of CO_2_–ionic liquid interfaces for the nucleation and aggregation centers of MOFs. By altering the CO_2_ pressure, it was simple to modify the morphology of the MOF. In the absence of CO_2_, only nanoscale aggregates without tetrahedral structures were produced. When CO_2_ pressure was low, there was a mixture of nanoscale aggregates and tetrahedrons. However, more tetrahedron particles formed under high CO_2_ pressure because more CO_2_ bubbles were generated under high pressure, providing more interfaces for the growth of MOFs. Maspoch et al. used spray–drying as a general method to assemble MOFs [48]. Without the use of any extra-incompatible solvents, surfactants, emulsifiers, or threads, spray–drying creates uniformly heated droplets of any preferred solvent containing the precursor. The precursor solution is first atomized into a spray of droplets using a two–fluid nozzle by injecting compressed air or nitrogen simultaneously at different rates. Therefore, each droplet that comes in contact with the gas stream is suspended and then heated to a temperature that allows the solvent to evaporate and causes the radial diffusion of the precursor towards the droplet surface. As the droplet evaporates, the droplet surface recedes causing the surface. The precursor concentration is increased until the critical concentration of the reaction is attained and the nano–MOF starts to crystallize on the surface. Maspoh et al. also successfully synthesized hollow HKUST–1 using this method. Copper nitrate and phthalic acid were spray-dried in a solution consisting of water, dimethylformamide, and ethanol using a small spray–dryer at a given feed rate and inlet temperature. The synthesized HKUST–1 had a hollow superstructure and could be separated into nanosized HKUST–1 crystals with an average dimension of 75 ± 28 nm under sonication. Additionally, a series of hollow MOFs including Cu–BDC [49], MIL–88A [50], MIL–88B [51], MOF–14 [52], MOF–74 [53], and UIO–66 [54] were prepared, demonstrating the versatility of this method. The liquid–liquid interface method is mainly utilized to prepare hollow MOFs by forming emulsion droplets as microreactors. For example, Xu et al. used water and n-octanol as water and oil phases, respectively (Figure 8) [55]. As Zn^2+^ was confined in the water phase and 2-methylimidazole disseminated in the oil phase, a ZIF–8 started to nucleate at the two-phase interface, forming a hydrophobic environment that was conducive to 2–MeIM’s diffusion from the oil phase to the interface and then into the water phase. Therefore, ZIF–8 crystals continued to grow inward. Hollow structures with varied shell thickness can be easily obtained by controlling the synthesis time. A striking feature of the emulsion–interfacial approach is that an abundance of hollow ZIF–8 spheres can be fabricated without templates and subsequent etching. In the solid–liquid interface approach, a metal precursor and organic linkers are in a liquid phase, and the contact area is constrained to the solid surface. These materials, including metals, metal hydroxides, or metal oxides, not only provide solid–liquid interface regions, but also act as sacrificial agents to form MOF crystals at the interface. The formation of hollow cavities is mainly based on the Kirkendall effect. Metal ions created by the solid metal precursor’s progressive dissolution are eventually coupled with the ligand to form a MOF shell when it is joined with the organic linker.

## 3. Applications

### 3.1. Electrochemical Sensing

Owing to the diversity in structure and composition of MOFs, their highly regulable chemical properties, and their facile interactions with guest molecules, the enrichment and removal of some target substances in MOFs can be accomplished simply and can lead to prominent electronic changes and a corresponding electrical response. Thus, MOFs have been a burgeoning platform for sensors that are able to detect and interact with many analytes, including heavy metal ions, biomolecules, toxic substances, and factory waste gases [56,57,58]. Nevertheless, the inherent shortcomings of MOF materials, including their poor electrical conductivity and tardy mass-transfer rate, limit their application in the field of electrochemical detection. To a large extent, pristine MOFs hinder the mass transfer of analytes on the material and affect the output of detection signals, which leads to great obstacles in their application in the field of electrochemical sensing. Meanwhile, the poor electrical conductivity of MOFs themselves further hinders the output of electrical signals, resulting in the inability to obtain high–sensitivity electrochemical sensors. 

**Figure 9:**
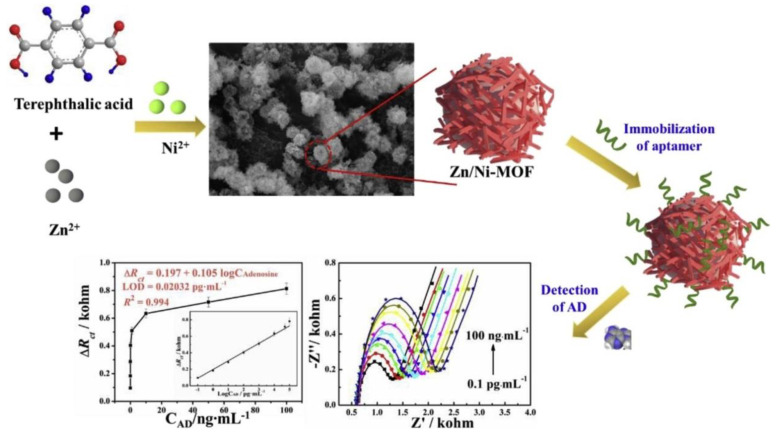
The synthesis process of a ZnNi MOF for AD detection. Reprinted with permission from Ref. [60]. Copyright 2019, Elsevier.

Hollow MOFs greatly enhance their mass-transfer capacity due to their unique hollow cavity and thin shell. Yao’s group fabricated MIL-101 hierarchical hollow cages and constructed an electrochemical sensor using hollow MIL-101 to measure nitrofuranone (NFZ) [59]. The sensor’s linear range was wide (0.030–55 M), while its detection limit was low (10 nM). After six parallel measurements, the recoveries remained in the range of 93.2–103%. The good detection performance of the sensor was ascribed to the huge specific surface field and quick electron-transfer rate. The internal cavity of the hollow MIL-101 was conducive to the aggregation of NFZ and provided more catalytically active sites. Similarly, Du et al. reported an unlabeled electrochemical adaptive sensor using a bimetallic, hollow MOF for the efficient detection of adenosine (Figure 9) [60]. Adenosine is a potential tumor marker and has key signal transmission functions in the nervous system. It has many important physiological and pharmacological functions, including the dilation of coronary vessels, the regulation of the immune response, and the inhibition of the inflammatory response when the myocardial oxygen supply is reduced or its load is increased. Therefore, the detection of AD is very important for clinical diagnosis. By altering the Zn^2+^/Ni^2+^ ratio, they prepared three kinds of hollow Zn/Ni–MOFs with different morphologies. When the Zn^2+^/Ni^2+^ ratio was 1:2, a Zn/Ni–MOF with a layered, hollow, microsphere structure was obtained. In this morphology, the immobilized number of aptamer chains was greatly increased, thereby facilitating AD binding. Bimetals can not only tune the morphology of MOFs but can also enhance their electrochemical properties and catalytic activity. The content of Ni^2+^ has an important effect on the anchoring of the aptamer chain because of the sturdy adsorption between the nickel center and –NH_2_. Thus, the layered structure and synergistic effect of nickel and zinc will help to improve the sensor property for AD.

**Figure 10:**
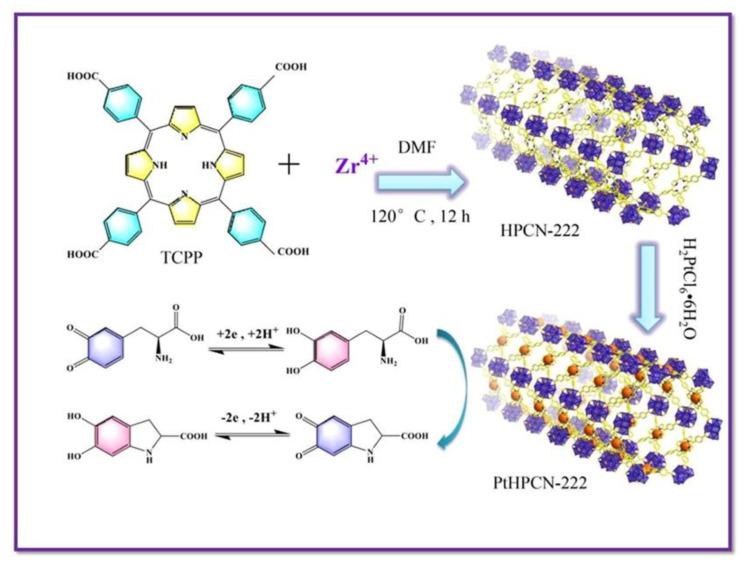
Preparation of PtHPCN–222. Reprinted with permission from Ref. [61]. Copyright 2022, Elsevier.

In addition, the combination of hollow MOFs with metals, metal oxides, and conductive polymers, etc. can solve the problem of poor conductivity. Liu et al. immobilized single platinum atoms on a hollow Zr–MOF (HPCN–222) for the electrocatalytic sensing of levodopa [61]. The linear range of the PtHPCN–222/GCE sensor was between 0.1–1 and 1–130 μM and had excellent recovery properties (Figure 10). The usage of Pt atoms was substantially enhanced by using the hollow PCN–222, with its high specific surface area, as the carrier of Pt atoms. In addition to increasing the substrate’s diffusion rate, PtHPCN–222’s hollow structure and wide surface area offered many redox-active sites that could interact with levodopa. Moreover, the load of Pt atoms provided more catalytic active centers and enhanced the response signal to the target.

### 3.2. Catalysis

As burgeoning porous materials, hollow MOFs have obtained extensive attention in catalysis. As a result of inheriting the merits of pristine MOFs with large surface areas and plenty of catalytic active centers, the intermediate cavities peculiar to hollow MOFs greatly shorten the diffusion path length of reactants and improve the catalytic performance. Therefore, hollow MOFs have great potential for organic catalysis with high activity, excellent selectivity, and fast kinetics. Liu et al. achieved the preparation of a hollow, mesoporous, hierarchical structure MIL–101 (HM–MIL) [44]. They carefully designed a bimetallic MOF with different metal–ligand bond stabilities and spatial distribution. By the enhanced nucleation process and selective etching treatment, hollow and mesoporous shells of MOFs were realized and used for catalytic reactions with 1–Chloro–4–ethenylbenzene as a probe. The void diameter and shell thickness of MIL–101 can be well adjusted by adjusting the response parameters. Benefiting from a higher porosity and wider channels, the mass-transfer resistance of guest molecules in HM–MIL was greatly reduced, and thus the transport rate was increased. Meanwhile, the rapid diffusion of 1–Chloro–4–ethenylbenzene in MOFs greatly improved their selectivity, as the rapid diffusion of 4-chlorostyrene in MOFs reduced their further oxidation. After five cycles, HM-MIL still exhibited excellent stability. Li et al. fabricated a hollow ZIF-8 as an excellent catalyst for cycloaddition reactions [62]. This kind of nano–catalytic carrier had a flexible shell and mesopore, which can effectively promote the regulation and diffusion of a matrix of various sizes. With 1, 3–cyclohexanedione and enal as the target compounds, the catalytic conversion and selectivity of the material to the target compounds reached 89% and 99.9%, respectively. Beyond that, the material could be utilized roughly ten times without losing any of its functionality. Zhang et al. constructed a Co–MOF–74 hollow structure for the first time with a thin shell measuring approximately 50 nm [63]. It also performed well for the thermocatalytic cyanosilication of aldehydes and the photocatalytic oxidation of thioanisole. This hollow Co–MOF–74 was composed of 8−18 nm nanoparticles and was different from its counterpart of an ordinary, three-dimensional structure. Such a structure had a greater availability of the active sites and an improved ability to capture light, and therefore exhibited excellent photocatalytic activity for thioanisole. When the reactants were catalyzed by the hollow CO–MOF–74, the benzene ring connected aldehydes with electron–drawing groups, which were more reactive than those with electron-donating groups. The aldehydes were activated by unsaturated Co, whispering the reactants. A similarly effective heterogeneous catalyst has been employed with a double-shell hollow MOF. For example, an Au/MOF possessing a double shell was successfully synthesized and used for the tandem catalytic synthesis of imines from benzyl alcohol and aniline under air atmosphere and solvent-free conditions [64]. The highest turnover frequency recorded was 170 h^−1^, and the conversion rate and selectivity were above 99%. It is well–known that hollow structures have been shown to enhance catalytic activity because of their characteristic properties in promoting mass transfer. The synergistic effect of hollow structures with Au is also crucial for the cascade catalytic reaction.

**Figure 11:**
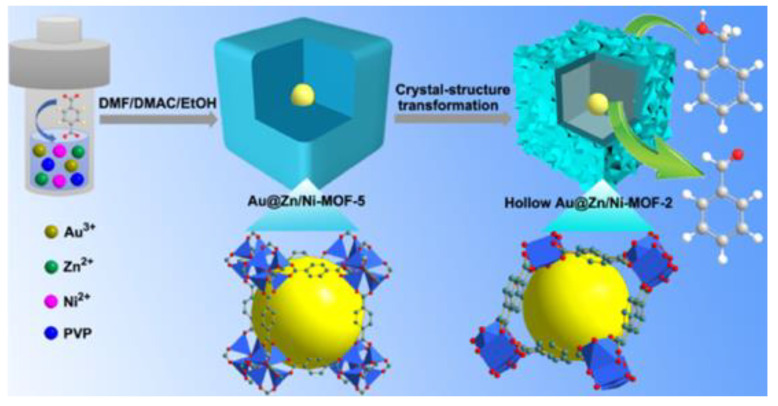
Fabrication of hollow Au@Zn/Ni–MOF–2. Reprinted with permission from Ref. [66]. Copyright 2021, American Chemical Society.

In order to achieve more catalytic effects, it has also become a trend to combine hollow MOFs with other active materials. For instance, a Zr/Ce, bimetallic, hollow-MOF nanoreactor was successfully synthesized by using a non-toxic and colloidal carbon template [65]. By adjusting the Ce/Zr ratio, the structure of nanospheres changed to some extent. When the Ce/Zr ratio was 1:4, the most ideal hollow structure was created, which was applied to accelerate the transformation of 2–furanaldehyde into 2–furylmethanol. The Lewis acid site of Zr(IV) in UIO–66 can activate furfural and transfer the dissociated hydrogen from the alkol to furfural to form furfural alcohol. However, with the Ce content increase, the activity of the composite decreased. This is because the Lewis acidity of Zr is higher than that of Ce. Therefore, excessive Ce doping will reduce the Lewis acidity of MOF and reduce its catalytic capacity. After testing the recovery performance of the catalyst, it was found that the conversion rate of furfural was maintained at 86% after three cycles, which proved that the material was relatively stable. Additionally, a new type of hollow core–shell nanoreactor also was proposed, which had Au and Zn/Ni–MOF–2 as the core and shell [66]. A hollow, bimetallic, MOF–embedded gold structure was fabricated using a simple, one–step strategy without employing a template for selective oxidizing alcohol. With methylbenzene and air as the solvent and environmental oxidant, respectively, the conversion rate of benzyl alcohol could be as high as 98% (Figure 11). Moreover, the selectivity of aldehyde and the conversion of benzyl alcohol did not change significantly after the reactor was reused five times, which proved that hollow Au@Zn/Ni–MOF–2 has good stability. Among them, small and well-distributed Au nanoparticles are the key to improve catalytic properties. The hollow Zn/Ni–MOF shell and Au nanoparticle core promoted the enhanced activity through a synergistic effect. In addition, bimetals were also used to be compounded with hollow MOFs for efficient catalysis. Oh et al. encapsulated highly active, bimetallic PdCo nanoparticles in a hollow ZIF–67 for the reduction of 4–nitrophenol. [67] Through the thermal decomposition of the PS@ZIF–67/Pd^2+^ under diverse temperatures, a hollow MOF and its derived carbon materials can be obtained. Pd^2+^ and Co^2+^ were successfully reduced to metal Pd and Co during pyrolysis. The hollow ZIF of the PdCo–loaded alloy revealed efficient catalytic activity for reducing 4–nitrophenol. The conversion from 4–nitrophenol to 4–aminophenol was completed within two minutes, and the TOF value was 57 min^−1^, which was more than that of the reported catalysts. This was not only due to the unique, hollow MOF skeleton, but also the excellent catalytic activity of the bimetal and the electronic effects between Pd and Co, which caused the bimetal to have better catalytic activity than a single metal.

In summary, the unsaturated metal sites, porous structures, and internal cavities of hollow MOFs can be applied in catalysis. In addition, within the loading or decoration of different external active components, hollow MOFs can show excellent performance in different catalytic systems.

### 3.3. Adsorption and Removal

**Figure 12:**
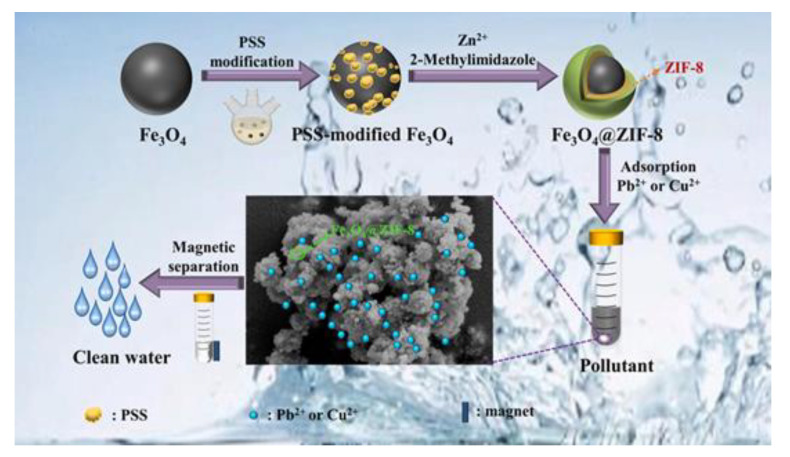
Preparation of Fe_3_O_4_@ZIF–8 for removing metal ions. Reprinted with permission from Ref. [71]. Copyright 2021, Elsevier.

In recent decades, the pollution of water and the discharge of toxic, polluting chemicals are becoming more and more serious, leading to a battery of ecological problems such as global climate change, harm to humans, and botany destruction. Therefore, the exploration of high-efficiency environmental treatment materials is imminent and has received extensive attention [68]. N, O, and other elements rich in the ligands of MOFs can effectively capture radionuclides and heavy metal ions through the coordination or ion–exchange reactions, and their empty cavities can reduce the mass–transfer resistance and improve the flux. For example, Zhang et al. prepared a neoteric uranium adsorbent, a diaminomaleonitrile (DAMN) –modified chromium terephthalate (III) double-shell hollow (DSHM) metal–organic skeleton, by using the post-synthesis method of coordinating an unsaturated site by grafting amino groups [69]. This was a new idea of using a hollow–MOF adsorbent to remove radioactive particles from seawater. The hollow MOFs have abundant exposed and accessible unsaturated metal sites, providing more chances for the coordination of –NH_2_ and –C=N with uranium ions. Adsorption experiments under simulated seawater conditions showed that DSHM–DAMN demonstrated a high selectivity for uranium, and the removal efficiency of uranium could still reach 85% in the presence of other particles. This is attributed to the use of a functionalized amino group as a hard base, which binds preferentially to uranium as a hard acid. Thus, DSHM–DAMN has a high affinity for uranium. Similarly, Cho et al. successfully used a ferrocyanide–functionalized ZIF–8 for efficiently and selectively removing radioactive Cs^+^ from wastewater [70]. After modification with FC, a ZIF–8 with a hollow structure was obtained. Due to a higher affinity between FC and Cs^+^, the composites had a high efficiency and selectivity for Cs^+^ removal, with a maximum adsorption capacity of 422.42 mg·g^−1^. By adjusting the FC/ZIF–8 ratio between 0.4 and 0.8, the maximum adsorption capacity also changed from 257.41 to 422 mg·g^−1^. At the same time, in a wide range of pH, the composite materials had a relatively stable adsorption of Cs+, and adsorption capacity was more than 400 mg·g^−1^. Therefore, ZIF–8–FC can adsorb radioactive Cs^+^ in various environments. In addition, Wang et al. synthesized a Fe_3_O_4_@ZIF–8 magnetic material and utilized it to adsorb heavy metal ions Cu^2+^ and Pb^2+^ (Figure 12) [71]. The adsorption of Cu^2+^ and Pb^2+^ on Fe_3_O_4_@ZIF–8 can reach equilibrium in 20 and 60 min, and the values of Q_max_ are 724.4 and 301 mg·g^−1^, according to the Langmuir model. Moreover, the composite material can be reused at least four times. With the exception of inorganic ions, organic molecules such as drugs can also be quickly and efficiently removed by adsorption. For instance, Zhang et al. synthesized a tannic–acid–etched ZIF–8/GO–hollow–composite membrane for adsorbing organics and salts for the first time [72]. The hollow ZIF–8 enlarged the interlamellar spacing of GO sheets, promoted the formation of water channels, and notably reduced the transfer resistance of water. The prepared material showed good performance in the disposition of wastewater, with a more than 99% repulsive rate of the composite film to Congo red dye. Furthermore, Li et al. prepared MOF–808 for the adsorption of the anti-inflammatory drug diclofenac [73] and used yttrium-stabilized zirconia hollow fiber as a scaffold, which is expected to be used commercially. In this study, the Q_max_ of diclofenac reached 833 mg·g^−1^; meanwhile, the removal rate was close to 95% within one hour. In practice, this structure solved the problem that the adsorbent was difficult to separate after the adsorption process finished. Additionally, the hollow fiber structure greatly improved the superficial area of adsorbents and solved the problem of uneven flow distribution, which was inevitable in the traditional, packed–bed adsorption tower. Similarly, Wang et al. combined a hollow Co–MOF–74 with electrospinning fiber membranes for removing polycyclic aromatic hydrocarbons by [74]. The composite material had strong hydrophobicity, outstanding mechanical properties, and, after adsorption, the filter and membrane were easy to recover when compared with powder MOFs. The synthesized, hollow nanofiber membranes maintained a high recovery rate after repeated reuse, and the adsorption capacity of seven PAHs reached 161–214 mg·g^−1^. All these results proved that H–NFMs had great application prospects in the field of water treatment. Surprisingly, not only single pollutants but also mixed, inorganic–organic liquid pollutants also can be effectively removed using hollow MOFs. Ma et al. prepared a multi-layer, coaxial MOF hollow tube through freeze-drying for removing inorganic–organic mixed liquid pollutants with a removal efficiency of 94% [75].

### 3.4. Other Applications

In addition to the above centralized applications, hollow MOFs also have a very wide range of other applications, such as for drug transport, electrochemical energy storage, electrocatalysis, and so on. 

In recent years, the application of hollow MOFs in drug transportation and disease treatment has been a growing trend. Since the pore size, pore volume, and pore structure of hollow MOFs can be easily regulated by changing reaction parameters or adding active substances, they can be used as a potential platform for material embedding. Liu et al. synthesized a hollow Fe–MOF–5–NH_2_ and grafted it with biomolecules [76]. The hollow Fe–MOF–5–NH_2_ was able to hold more drugs, with a drug load of up to 35%, benefiting from its hollow structure. Drug release can be regulated by pH changes for more precise cancer treatment. Additionally, Jiang et al. prepared TCBPE–based nanotubes for the first time with a hollow structure that were capable of emitting intense fluorescence [77]. The embedded adriamycin anticancer medication was supplied in a self-directed manner, and this hollow MOF structure was very biocompatible and photostable. Drug loading is substantially higher than with pristine MOFs, reaching up to 36%, thanks to their hollow structure and strong π–π stacking. Similarly, the release of adriamycin is pH dependent. Hollow MOFs have been used as electrode materials due to their inorganic–organic hybrid properties and adjustable structure. However, the inherent poor conductivity of MOFs prevents their application in batteries to some extent. In general, this problem can be effectively solved by adding conductive materials. For example, Hao et al. reported a hollow, Co–MOF–74, porous structure composed of super-fine nanomaterials that was used as an anode for lithium-ion batteries [78]. By adjusting the reaction parameters to optimize the material, the optimized hollow Co–MOF–74 had a large specific surface area, coordination defects, and a hierarchical porous structure, which was conducive to lithium storage. It had a high reversible capacity (820 mAh g^−1^ at 1000 ma g^−1^) and circulatory stability (996 mAh g^−1^ at 1000 mA g^−1^ after 470 cycles). Supercapacitors also attract great attention in the field of energy storage because of their advantages, such as fast charging and high energy density. Research on simple and high–performance supercapacitors has been in progress. Among many candidate materials, hollow MOFs have great application potential. Han et al. combined transition metal oxides with hollow MOFs as electrode materials for self-sustaining batteries, and the synthesized Co–MOF@CoCr_2_O_4_ had a high specific capacity and excellent cyclic stability [79]. As for electrocatalysis, hollow–structure MOFs can effectively increase the reaction of active sites and improve the mass-transfer efficiency due to their abundant exposed active sites and large internal cavities, thus benefiting the electrocatalytic performance. Gu et al. reported a novel, in situ, simultaneous empty–doping method for the construction of bimetallic, doped, CoM–MOF hollow nanospheres for electrochemical hydrogen production [80]. Due to its rigid structure and the synergistic effect between the cavity and the bimetal, the charge–transfer resistance of the composite is only 42 Ω, and the phase-education of the solid Co–MOF has better electrocatalytic performance. In addition, hollow–MOF–derived materials have also been used in the field of electrochemical conversion [81,82,83,84]. By adjusting the morphologies of MOFs, the resultant materials can produce superior electrocatalytic properties in terms of energy conversion and storage applications.

## 4. Summary and Outlook

This article sums up the hollow–MOF synthesis methods and their applications in chemical catalysis, electrochemical sensing, and adsorption removal. Synthesis strategies for hollow MOFs can be divided into template template-free methods, according to the existence of templates. These methods have their own advantages and disadvantages for the preparation of hollow MOFs. Despite significant advancements in the synthesis of hollow MOFs, their inherent disadvantages—such as poor electrical conductivity and poor stability—still hinder their applications, especially in electrochemical sensing. Therefore, based on the preparation of hollow MOFs, the above problems can be solved in some ways as a carrier by compounding with functional materials, including metal nanoparticles, metal oxides, and polymers. Looking towards the future, it is expected that the research will explore more convenient, efficient, and versatile synthesis strategies for hollow MOFs and their composites. Hollow MOFs and their composite materials are also expected to participate in more advanced areas such as drug delivery, photocatalysis, and water remediation because of their flexible and regulable structures, excellent catalytic activity, and biocompatibility. Additionally, hollow MOFs are considered to be an excellent precursor material for synthesizing hollow carbon materials and deuterogenic hollow metal oxides. These materials not only inherit the advantages of the original hollow MOFs, but also have better conductivity and stability, so they have extensive practical applications in electrochemical energy areas.

## Figures and Tables

**Table 1:** A list of abbreviations used in this review article.

Abbreviation	Meaning
AD	Adenosine
ARS	Alizarin red S
CMC	Critical micelle concentration
CNT	Carbon nanotube
CnTAB	Alkyltrimethylammonium bromide
DAMN	Diaminomaleonitrile
DMAC	Dimethylacetamide
DMF	N,N–Dimethylformamide
GA	Gallic acid
GO	Graphene oxide
LDH	Layered double hydroxide
MIL	Materials of Institute Lavoisier
MO	Methyl orange
MOFs	Metal–organic frameworks
NaDC	Metal–sodium deoxycholate
NFZ	Nitrofuranone
NPs	Nanoparticles
PAHs	Polycyclic Aromatic Hydrocarbons
PS	Polystyrene
SDS	Sodium dodecyl sulfate
TA	Tannic acid
TOF	Turn over frequency
UIO	University of Oslo
ZIF	Zeolitic imidazolate framework

## Data Availability

Data are contained within the article.
